# Supplementary material for: Dengue during pregnancy and live birth outcomes: a cohort of linked data from Brazil
Source: BMJ Open. 2019 Jul 24;9(7):e023529. doi: 10.1136/bmjopen-2018-023529 (PMC6661644; doi:10.1136/bmjopen-2018-023529)
Supplement: Author's manuscript [file bmjopen-2018-023529.draft_revisions.pdf]

BMJ Open is committed to open peer review. As part of this commitment we make the peer review history of every article we publish publicly available.

When an article is published we post the peer reviewers' comments and the authors' responses online. We also post the versions of the paper that were used during peer review. These are the versions that the peer review comments apply to.

The versions of the paper that follow are the versions that were submitted during the peer review process. They are not the versions of record or the final published versions. They should not be cited or distributed as the published version of this manuscript.

BMJ Open is an open access journal and the full, final, typeset and author-corrected version of record of the manuscript is available on our site with no access controls, subscription charges or pay-per-view fees (http://bmjopen.bmj.com).

If you have any questions on BMJ Open's open peer review process please email info.bmjopen@bmj.com

BMJ Open

Dengue during pregnancy and adverse birth outcomes: a cohort analysis using routine data

Journal:	*BMJ Open*
Manuscript ID	bmjopen-2018-023529
Article Type:	Research
Date Submitted by the Author:	11-Apr-2018
Complete List of Authors:	Paixão, Enny; London School of Hygiene and Tropical Medicine Faculty of Epidemiology and Population Health, IDE<br>Campbell, Oona; London School of Hygiene and Tropical Medicine Faculty of Epidemiology and Population Health, IDE<br>Teixeira, Maria; Universidade Federal da Bahia<br>Costa, Maria Conceição; Federal University of Bahia, Instituto de Saúde Coletiva<br>Harron, Katie; London School of Hygiene and Tropical Medicine, Department of Health Services Research and Policy<br>Barreto, Mauricio; Instituto de Saude Coletiva/UFBa,<br>Leal, Maira; Universidade Federal da Bahia<br>Almeida, Marcia; Universidade de São Paulo, School of Public Health<br>Rodrigues, Laura; London School of Hygiene and Tropical Medicine,
Keywords:	Dengue, pregnancy, birth outcomes

SCHOLARONE™
Manuscripts

For peer review only

Dengue during pregnancy and adverse birth outcomes: a cohort analysis using routine data

Running title: Dengue and adverse birth outcomes

Enny S Paixão*, MSc[abc]
Oona M.R.Campbell, PhD[a]
Maria Glória Teixeira, PhD[bc]
Maria da Conceição N. Costa, PhD[b]
Katie Harron, PhD[a]
Mauricio L. Barreto,PhD[bc]
Maira B. Leal[b]
Marcia Furquim de AlmeidaPhD[d]
Laura C. Rodrigues, PhD[ac]

* Corresponding author email: npaixaoenfo@yahoo.com.br; Phone 44 02079588171

a London School of Hygiene and Tropical Medicine. Keppel St, Bloomsbury, London WC1E 7HT, United Kingdom

b Institutode Saúde Coletiva. Rua Basílio da Gama, s/n.Canela. CEP 40110040. Salvador, Bahia, Brasil.

c Center of Data and Knowledge Integration for Health (CIDACS), Instituto Gonçalo Moniz, Fundação Oswaldo Cruz, CEP 41745-715 Salvador-Bahia, Brazil

d Universidade de São Paulo.Av. Dr. Arnaldo, 715 Cerqueira Cesar, 01246904 - São Paulo, SP – Brasild

For peer review only

Abstract

Objectives: Dengue is the most common viral mosquito-borne disease, and women of reproductive age who live in or travel to endemic areas are at risk. Little is known about the effects of dengue during pregnancy on birth outcomes. The objective of this is study is to examine the effect of maternal dengue severity on outcomes.

Design and setting: We conducted a population-based cohort study using routinely-collected Brazilian data from 2006-2012.

Participating: We used birth registrations to identify pregnancies and linked to identify mothers of live births with dengue notified during pregnancy. It was included 16,738,00 live births. Using multinomial logistic regression and Firth method, we estimated risk and odds ratios for preterm birth, low birth weight and small for gestational age associated with dengue. We also investigated the effect of time between the onset of the disease and the outcome.

Results: We included 16,738,000 live births. Dengue haemorrhagic fever doubled the risk of preterm birth OR=2.4;95%CI 1.3-4.4 and low birth weight OR=2.1;95%CI 1.1-4.0 and did not have an effect on small for gestational age OR=2.1;95% CI 0.4-12.2). The magnitude of the effects was higher in the acute disease period.

Conclusion: This study showed an increased risk of adverse fetal outcomes in women with severe dengue during pregnancy. It appears that one contributing factor may be medical intervention to mitigate maternal risk with severe acute dengue.

Strengths and limitations of this study

- This is the first study to investigate the effect of dengue disease severity and time between the disease onset on preterm birth and low birth weight.

For peer review only

- This is the first study to investigate the risk of small for gestational age among women with dengue during pregnancy using a population-based approach with a sufficiently large sample size and controlling for confounders.
- This study relies on the use of secondary data, therefore outcome and exposure identification is susceptible to misclassification.
- The linkage process was rigorously validated and the results showed low sensitivity; however, it is unlikely that the linkage process introduced bias, since missed and false matches occurred randomly.

For peer review only

Introduction

Dengue is the most common viral vector-borne disease worldwide, with an estimated 390 million people infected each year, 96 million of whom will develop clinical symptoms. It is endemic in over 100 countries (mostly in South and Central American and Southeast Asia), and is spreading to new areas; according to the World Health Organization (WHO), approximately half of the world's population is at risk [1]. Since women of reproductive age who live in or travel to endemic areas are at risk of dengue, and since the effect of this disease during pregnancy is still unclear, it needs to be investigated. The majority of studies published to date are cases series[2]; the seven studies published before 2016 with comparison groups show conflicting results [3 4 5 6 7 8 9] and a meta-analysis published in 2016 found dengue during pregnancy was only associated with low birth weight and preterm birth in symptomatic women[2]. In 2017, a large Brazilian study found that notification of mild dengue during pregnancy was not associated with higher rates of preterm birth or lower birth weight in neonates compared to rates in a random sample of all neonates. However, the study found an increase in the risk of preterm birth among women with confirmed dengue compared with reported maternal dengue cases with a dengue negative test[10].

Maternal dengue, especially severe dengue, has been associated with fetal deaths[11] but the effects on those who survive, especially when the mothers progress to severe disease, needs to be better investigated. We analysed a large, population-based, retrospective cohort to investigate the association between symptomatic dengue notified during pregnancy and adverse fetal outcomes, and to examine the effect of maternal dengue severity on outcomes.

Methods

For peer review only

We conducted a population-based retrospective cohort study by linking routine records of live births with records of women notified and confirmed with dengue disease, in Brazil from January 1, 2006 until December 31, 2012.

Data sources

We extracted routinely collected data from two Brazilian databases.

1) The Live Births Information System (Sistema de Informação sobre Nascimentos; SINASC), which records live births in Brazil; these data are derived from the birth registration, a legal document completed by the health worker who attended the birth. It includes information on the mother (name, place of residence, age, marital status, education); the pregnancy (length of gestation, mode of delivery); and the neonate (birth weight, presence of congenital anomalies) [12]. Data completeness is very high, with 97% of Brazilian births registered,[13] and most variables were >90% complete[14].

2) Notifiable Diseases Information System (Sistema de Informação de Agravos de Notificação; SINAN), which records notifiable diseases. Dengue notifications information on the individual (name, place of residence, age, sex, and years of education), symptoms of the disease, laboratory tests, and disease severity, assessed clinically and laboratory according with the Brazilian dengue manual [15]. These data are reasonably complete on the variables used for linkage: <0.05% records were excluded because of a missing name. Around 5% of dengue cases did not have a final classification of severity. Laboratory confirmation was not required for notification or confirmation of dengue. During the study period (2006-2016), dengue was the main (and sometimes the only) vector-borne disease circulating in Brazil, as yellow fever and malaria occurred in restricted areas, and Zika and Chikungunya did not begin to circulate until 2014.

Procedures

For peer review only - http://bmjopen.bmj.com/site/about/guidelines.xhtml

SINASC defines a live birth as the product of conception that, independent of the duration of pregnancy, breathes or shows any other signs of life (such as a heartbeat, umbilical cord pulsation, or definite movement of voluntary muscles), after the separation from the mother's body [12]. Live birth records with a missing or implausible name of the mother (e.g. the name of a hospital as the name of the mother, twin or other multiple pregnancies, or congenital anomalies, or coming from municipalities without dengue notifications, and those with uncertain link status) were excluded (Figure 1).

Outcomes

We assessed three birth outcomes: preterm birth (<37 weeks); low birth weight (<2500g) and small for gestational age), calculated according to the Intergrowth scale [16] using the weight and gestational age in weeks at birth. This scale is sex specific, and those babies with a birth weight less than the 10th centile were considered small for gestational age. The data to calculate this outcome were available only for 2011 and 2012 (prior to this, gestational age was categorized).

Exposure

The exposure was being a confirmed case of dengue notified to SINAN during a pregnancy that resulted in a live birth. In Brazil, confirmation of dengue is based either on clinical and epidemiological criteria, namely presence of clinical symptoms of dengue in the same area and time as other confirmed cases of dengue, or on clinical and laboratory criteria, namely the presence of clinical symptoms and a positive test for one of: a) IgM detection by ELISA, b) viral RNA detection via PCR, c) NS1 viral antigen detection, or d) positive viral culture. Laboratory confirmed cases used in the analyses were referred to as “dengue during pregnancy, laboratory confirmed”. The notification does not necessarily state the woman notified was pregnant – there is a field for this variable, but it is largely missing. Rather, we considered a woman to be a case of dengue in pregnancy, and her newborn to be “exposed”,

For peer review only

if the woman was notified to SINAN and confirmed as a dengue case and she linked to a live birth in the nine months after notification. We used the clinical classification valid in Brazil in the period in which data were collected, which classified dengue into three clinical categories: a) “dengue fever”, b) “complicated dengue”, and c) “dengue haemorrhagic fever/ dengue shock syndrome”. a) Dengue fever is a self-limiting disease, characterized as fever, with a severe headache, pain behind the eyes, muscle and joint pain, and rash; b) Dengue haemorrhagic fever follows the WHO criteria and is characterized by fever, haemorrhagic evidence, thrombocytopenia and evidence of plasma leakage; c) Complicated dengue is a probable case of dengue characterized by one of the following: severe changes in the nervous system, cardiorespiratory dysfunction; insufficient hepatic function; gastrointestinal bleeding, cavity spills, or thrombocytopenia equal or less than 50,000/mm^3, or leucometry less than 1000/mm.[17]. Complicated dengue is a Brazilian definition for severe cases of dengue that do not meet WHO criteria for dengue haemorrhagic fever and cannot be classified as mild self-limited disease due to their severity;

Linkage process

The data used in the linkage process were identified because we probabilistically linked records of women with confirmed dengue (SINAN) with records of live births (SINASC) up to nine months after the SINAN notification, to identify those mothers who had dengue during pregnancy. For this, we used name, age or date of birth, and the place of residence of the mother at the time of delivery and of notification. We excluded records with missing or implausible names (Figure 1). Match weight calculations used the Fellegi-Sunter method.[18] Linkage followed the same procedure as previously described for linking stillbirth records and the dengue database in Brazil, in which we demonstrated a sensitivity of 62% and a positive predictive value of 95%.[19] To estimate parameters for linkage weights and to validate the quality of the linkage, we created a gold-standard dataset. For the dengue-live birth

For peer review only - http://bmjopen.bmj.com/site/about/guidelines.xhtml

linkage, we used all live birth records in Ceará and Espirito Santo States for 2010. Record pairs were manually reviewed to establish true matches (records belonging to the same mother) and to derive match weights. Records pairs were then ordered by match weight and manually inspected to identify any errors in linkage (false matches or missed matches). Live birth records linked to confirmed dengue notifications with high certainty were classified as dengue in pregnancy cases, and birth records we were confident had no link were classified as non-dengue cases. Uncertain links (where we could not establish whether a woman had dengue) were excluded to avoid misclassification.

Statistical analysis

The data used in the analysis were de-identified. We estimated the crude and adjusted risk ratios from multinomial logistic regression, controlling for maternal age (categorized as <=20; between 20-35 and >=35 years old), education (less than 3 years; between 4-7 years and more than 8 years) and marital status (single/ widow /divorced or married/stable union). For a sensitivity analysis of the validity of clinical/epidemiologic diagnosis, we repeated the analyses using laboratory confirmed dengue only. We investigated the effect of time between disease onset and live birth outcomes of preterm birth, low birth weight, and small for gestational age. In general, dengue is an acute disease with rapid recovery. The time between disease onset and the outcome was calculated using the date of the disease onset (information available in SINAN) and the date when the outcome occurred (date of live birth); we categorized this difference as being less than or equal to ten days or greater than 10 days. For the analyses by dengue severity (mild dengue, dengue with complications and dengue haemorrhagic fever), we calculated the odds ratio because we used the Firth method (to reduce the small sample bias in maximum likelihood estimation) as we were analysing rare events, and controlled for maternal age, education and marital status [20].

Patient and Public Involvement

For peer review only

There was no patients involved in the research. The data used are administrative routinely collected data.

Ethics Statements

Ethical approval was obtained from The Federal University of Bahia, Salvador, Brazil (CAAE: 26797814.7.0000.5030) and from The London School of Hygiene and Tropical Medicine (Ethics Ref:10269).

For peer review only

Results

The SINASC recorded 20,333,482 live births from 2006-2012. After exclusions, 16,738,000 live births were included in the study, and 17,673 (0.1%) of their mothers were linked with a dengue notification record (Figure 1).

The cohort characteristics by dengue status are described in Table 1. Compared to pregnant women without dengue, pregnant women with dengue were more likely to have more years of formal education and to have delivered by caesarean-section.

Table 1. Maternal characteristics, delivery details, and birth outcomes in relation to dengue status, Brazil, 2006-2012

Characteristics	Notified with confirmed dengue in pregnancy n (%)	Without dengue notification n (%)	p value
**Age of the mother**			
< 20	4,499 (25.5)	4,384,159 (26.2)	<0.001
20-35	11,981 (67.8)	11,042,342 (66.0)	
>35	1,193 (6.7)	1,293,171 (7.8)	
Missing	-	655 (0.0)	
**Maternal education**			
Less than 3 years	1,065 (6.1)	1,358,815 (8.3)	<0.001
4-7 years	4,661 (27.0)	4,592,979 (28.1)	
More than 8 years	11,578 (66.9)	10,399,703 (63.6)	
Missing	369 (2.1)	368,830 (2.2)	

Characteristics	Notified with confirmed dengue in pregnancy n (%)	Without dengue notification n (%)	p value
**Marital status**			
Single/Widow/Divorced	11,518 (66.1)	9,660,306 (58.7)	<0.001
Married /Union	5,899 (33.9)	6,797,658 (41.3)	
Missing	256 (1.4)	262,363 (1.6)	
**Numbers of pre-natal visits**			
Inadequate (less than 7 times)	6,991 (39.9)	6,817,679 (41.2)	<0.001
Adequate (7 or more times)	10,515 (60.1)	9,712,338 (58.8)	
Missing	167 (0.9)	190,310 (1.1)	
**Delivery**			
Vaginal	8,332 (47.2)	8,383,714 (50.2)	<0.001
C-section	9,320 (52.8)	8,307,072 (49.8)	
Missing	21 (0.1)	29,541 (0.2)	
**Gestational age in the delivery**			
Less than 22 weeks	17 (0.1)	8,844 (0.0)	<0.001
22-27 weeks	68 (0.4)	58,760 (0.4)	
28-31 weeks	147 (0.8)	112,407 (0.7)	
32-36 weeks	1,157 (6.7)	1,011,633 (6.1)	
More than 37 weeks	15,994 (92.0)	15,215,440 (92.8)	
Missing	290 (1.6)	313,243 (1.9)	
**Birth weight**			
>=3,000	12,171 (69.1)	11,659,487 (69.9)	<0.001
3,000-2,500	3,973(22.5)	3,816,201 (22.9)	
1,500-2,500	1,258 (7.1)	1,026,248 (6.1)	
<1,500	226 (1.3)	178,643 (1.1)	
Missing	38 (0.2)	39,748 (0.2)	
**Small for gestational age (10th centile)$^{\#}$**			
Normal	3,681 (91.7)	3,289,125 (92.0)	0.608
Small	331 (8.2)	287,173 (8.0)	
Data not collected	13,661 (77.3)	13,144,029 (78.6)	

*% of each category without the missing value
# data available only in 2011 and 2012

Maternal dengue slightly increased the risk of preterm birth from 7.3% to 7.9%, RR 1.1 (95% CI 1.0-1.2) and low birth weight from 7.2% to 8.4% RR1.2 (95% CI 1.1-1.2), although the

For peer review only

confidence interval was borderline. There was no association between maternal dengue and being small for gestational age (Table 2). Restricting analyses to 33%, (5,755/17,388) of laboratory confirmed dengue cases did not change the magnitude of the associations.

Table 2. Number of cases notified with dengue during pregnancy (N), risk ratio crude and adjusted for the association among dengue during pregnancy and preterm birth, low birth weight, small for gestational age. Brazil, 2006-2012.

	All dengue cases		Lab confirmed dengue cases	
**Outcomes**	**Crude Risk Ratio (95 % Confidence Interval)**	**Adjusted Risk Ratio (95 % Confidence Interval)**	**Crude Risk Ratio (95% Confidence Interval)**	**Adjusted Risk Ratio (95% confidence Interval)**
**Preterm birth N=1,193,033**				
	N= 1,389 (0.12%)		N=439 (0.04%)	
Overall (<37 weeks)	1.1 (1.0-1.2)	1.1 (1.0-1.2)	1.1 (1.0-1.2)	1.1 (1.0-1.2)
Less than 28 weeks	1.1 (1.0-1.1)	1.1 (1.0-1.2)	1.0 (0.9-1.2)	1.0 (0.9-1.2)
28-32 weeks	1.2 (1.1-1.5)	1.2 (1.1-1.4)	1.2 (0.9-1.6)	1.2 (0.9-1.6)
32-36 weeks	1.2 (1.0-1.5)	1.1 (0.9-1.4)	1.1 (0.7-1.6)	1.1 (0.7-1.6)
**Low birth weight N=1,206,375**				
	N=1484 (0.12%)		N=475 (0.04%)	
Overall (<2500g)	1.2 (1.1-1.2)	1.2 (1.1-1.2)	1.2 (1.0-1.3)	1.2 (1.1-1.3)
1500g-2499g	1.2 (1.1-1.2)	1.2 (1.1-1.2)	1.2 (1.0-1.3)	1.2 (1.1-1.3)
Less than 1500g	1.2 (1.1-1.4)	1.2 (1.0-1.3)	1.1 (0.9-1.4)	1.1 (0.9-1.4)
**Small for gestational age N=287,504**				
	N=331 (0.12%)		N=68 (0.02%)	
Overall (<10th centile)	1.0 (0.9-1.1)	1.0 (0.9-1.1)	0.9 (0.7-1.1)	0.9 (0.7-1.2)

Adjusted for maternal age, education, marital status

Table 3 shows the dose-response effect of the severity of dengue on preterm birth, low birth weight and small for gestational age. Dengue haemorrhagic fever in pregnancy doubled the odds of preterm birth from 1,191,644/16,407,084 (7.3%) to 12/79 (15.2%), OR 2.4 95% CI 1.3-4.4) and of low birth weight from 1,204,891/16,680,579 (7.2%) to 11/79 (13.9%), OR 2.1

For peer review only

For peer review only - http://bmjopen.bmj.com/site/about/guidelines.xhtml

95% IC 1.1-4.0), but we did not see a significant effect of dengue on small for gestational age even among pregnant women who developed haemorrhagic disease (from 287,173/3,576,298 (8.0%) to 1/9 (11.1%), OR 2.5 95% CI 0.4-12.2).

Table 3. Number of cases with dengue during pregnancy (N) and Odds Ratio for the association Dengue during pregnancy by severity of disease and adverse birth outcomes (preterm birth, low birth weight, small for gestational). Brazil, 2006-2012.

Outcome	Mild dengue Odds Ratio (95 % Confidence Interval)	Complicated dengue Odds Ratio (95 % Confidence Interval)	Haemorrhagic fever Odds Ratio (95% Confidence Interval)
**Preterm birth (<37 weeks) N=1,192,920**			
N (%)	1,234 (0.103%)	30 (0.002%)	12 (0.001%)
Crude	1.1 (1.0-1.1)	1.4 (0.9-1.9)	2.4 (1.3-4.3)
Adjusted	1.1 (1.0-1.1)	1.4 (0.9-2.0)	2.4 (1.3-4.4)
**Low birth weight (<2500g) N=1,206,265**			
N	1,327 (0.110%)	36 (0.003%)	11(0.001%)
Crude	1.1 (1.1-1.2)	1.6 (1.2-2.3)	2.1 (1.1-4.0)
Adjusted	1.1 (1.1-1.2)	1. 6 (1.1-2.3)	2.1 (1.1-4.0)
**Small for gestational age (10th centile) N=287,474**			
N	294 (0.102%)	6 (0.002%)	1(0.000%)
Crude	1.0 (0.9-1.1)	2.2 (0.9-5.1)	2.0 (0.3-11.4)
Adjusted	1.0 (0.9-1.1)	2.3 (1.0-5.3)	2.1 (0.4-12.2)

Adjusted for maternal age, education, marital status

***Because of the small numbers, we included extra decimal places.**

The risk of dengue in pregnancy on fetal outcomes depended on the time between dengue onset and the date of live birth: the magnitude of the effect of dengue on adverse birth outcomes was higher during the acute disease period, with some residual effects remaining after the first 10 days for preterm birth and low birth weight (Table 4).

For peer review only

Table 4. Number of cases with dengue during pregnancy (N) and risk Ratio for the association Dengue during pregnancy by timing of disease and adverse birth outcomes (preterm birth, low birth weight, small for gestational). Brazil, 2006-2012.

Outcome		Risk Ratio and frequency of outcomes within 10 days of disease onset (95 % Confidence Interval)	Risk Ratio and frequency of outcomes after 10 days from disease onset (95 % Confidence Interval)
**Preterm birth**			
**N=1,191,719**			
	N	75 (0.006%)	1,314 (0.110%)
	Crude	2.1 (1.7-2.7)	1.1 (1.0-1.1)
	Adjusted	2.0 (1.6-2.6)	1.1 (1.0-1.1)
**Low birth weight N= 1,206,375**			
	N	74 (0.006%)	1410 (0.117%)
	Crude	2.1 (1.6-2.6)	1.1 (1.1-1.2)
	Adjusted	2.0 (1.6-2.6)	1.1 (1.1-1.2)
**Small for gestational age (10th centile) N=287,173**			
	N	4 (0.001%)	83 (0.029%)
	Crude	0.5 (0.2-1.5)	1.0 (0.9-1.1)
	Adjusted	0.5 (0.2-1.5)	1.0 (0.9-1.1)

Adjusted for maternal age, education, marital status

***Because of the small numbers, we included extra decimal places.**

Discussion

Cases of dengue haemorrhagic fever in pregnancy doubled the risk of preterm birth (OR: 2.4) and of low birth weight (OR: 2.1); by contrast the effect of mild dengue fever only raised the risk by 10 to 20 percent for preterm birth and low birth weight respectively. There was no increase in the risk of small for gestational age for either severe or mild disease. The main

For peer review only

dengue effect on birth outcomes occurred during acute disease, within the first ten days of disease onset.

Our results are consistent those with Nascimento, et al.[10] who showed that symptomatic dengue infection did not greatly affect the risk of low birthweight (1.00 95% CI 0.85-1.17) or preterm birth (OR: 0.98 95% CI 0.83-1.16) when compared to a random sample of live births. However, dengue has been associated with an increased risk of stillbirth, and by excluding fetal deaths in our analyses, we may be underestimating the risk by only considering birth outcomes in those who survived. Our findings are also consistent with a meta-analyses published in 2016,[2] which found an association between symptomatic dengue and preterm birth and low birth weight within in a hospital based population that sought care, and was likely to include only severe dengue cases. In our study, the association between maternal dengue and fetal outcomes was strongest for women with dengue haemorrhagic fever in pregnancy.

There is evidence that different infectious diseases in pregnancy can lead to adverse outcomes. The evidence is still fragmented, and there is no consistent effect, which appears to vary according to the pathogen involved, and the timing and severity of maternal disease. For instance, studies comparing pregnant women with infection to pregnant women without, found a) severe influenza increased the risk of preterm birth from 2.4 to 4 times, whereas studies based on mild range of illness did not find an effect; [21] b) measles and HIV infection increased the risk of low birth weight by 3.5 and 1.6 times, respectively. [22 23]

Our data suggest that the main way gestational dengue affects preterm birth and low birth weight is through maternal illness, rather than through a direct effect on the fetus. Since we did not observe an effect on small for gestational age, the effect of dengue in pregnancy on birth weight probably occurred via prematurity of the newborn. Therefore, much of the effect may be explained by early delivery due to medical interventions, such as caesarean section,

For peer review only

required because of concern about risk to the mother. The risk of prematurity was higher during the first 10 days after disease onset and among those with haemorrhagic disease. However, data on small for gestational age was only available for 2011-2012, which may have affected our ability to observe an effect.

The potential study limitations are associated with the linkage process and use of secondary data. In relation to the use of secondary data, outcome and exposure identification is susceptible to misclassification, a limitation inherent to this study design. Estimated national prevalence of preterm birth and small for gestational age in low and middle income countries using the available routine collected data are underestimated, however this error probably affected either, those exposed and no exposed to dengue therefore unlikely to bias the results of this study. [24, 25] Regarding the dengue diagnosis, it is usual practice in outbreaks/ epidemics to test until the origin of the outbreak is clearly established, and after that only test when there is a clinical indication. The proportion of laboratory confirmed cases among general population in Brazil is around 30%, [26] and therefore misclassification based on the lack of laboratory confirmation is possible but we showed very good agreement with the estimates of risk in subsets of the data with and without laboratory confirmation of dengue. The linkage was rigorously validated.[19] The results of this showed that despite the low sensitivity (that kept us from making statements about the prevalence of dengue during pregnancy) it is unlikely that the linkage process introduced bias, since missed and false matches occurred randomly. Although we adjusted for confounders, other possible unknown confounders, such as maternal co-morbidities or quality or type of obstetric care, may have contributed to the association between severe dengue and adverse fetal outcomes.

In summary, this study shows a more than doubling in the risk of preterm birth and low birth weight in women with dengue haemorrhagic fever in pregnancy, and increases of 10-20% in women with mild dengue. One contributing factor to adverse fetal outcomes may be medical

For peer review only

interventions triggered to mitigate mothers risk associated with dengue haemorrhagic fever. We recommend further research in different settings to confirm our results, and additional studies of adverse birth outcomes for other vector-borne diseases.

References

1. World Health Organization. Dengue and severe dengue. WHO Fact Sheet No117 Updated. April. 2017. http://www.who.int/mediacentre/factsheets/fs117/en/ (Accessed May 4, 2017).

2. Paixao ES, Teixeira, MG, Costa, MCN, Rodrigues LC. Dengue during pregnancy and adverse fetal outcomes: a systematic review and meta-analysis. Lancet Infect. Dis. **2016**;16:857-865.

3. Friedman EE, Dallah F, Harville EW et al. Symptomatic dengue infection during pregnancy and infant outcomes: a retrospective cohort study. PLoS Negl Trop Dis **2014**; **8**: e3226.

4. Tan PC, Rajasingam G, Devi S, Omar SZ. Dengue infection in pregnancy: prevalence, vertical transmission, and pregnancy outcome. Obstet Gynecol **2008**; 111: 1111–17.

5. Restrepo BN, Isaza DM, Salazar CL, et al. Dengue y embarazoen Antioquia, Colombia. Revista Facultad Nacional de Salud Pública **2004**; 22: 7–14.

6. Alvarenga CF, Silami VG, Brasil P, Boechat MEH, Coelho J, Nogueira RMR. Dengue during pregnancy: a study of thirteen cases. Am J Infect Dis **2009**; 5: 6.

7. Angarita LCR, Angarita SV, Correa M, Odreman MI. Transmisión perinatal del virus dengue en el binomiomadre-hijo. Arch Venez Pueric Pediatr **2003**; 76: 99–104.

For peer review only

8. Barroso LR, Betancourt ID, Saeta YF, Navarro MM, Guerra GD. Repercusión del dengue serotipo 3 sobre el embarazo y producto de la concepción. Rev Cuba Obstet Ginecol **2010**; 36: 42–50

9. Leite RC, Souza AI, Castanha PM, et al. Dengue infection in pregnancy and transplacental transfer of anti-dengue antibodies in Northeast, Brazil. J Clin Virol **2014**; 60: 16–21.

10. Nascimento LB, Siqueira CM, Coelho GE, Siqueira JB. Symptomatic dengue infection during pregnancy and livebirth outcomes in Brazil, 2007–13: a retrospective observational cohort study. Lancet Infect Dis. **2017**;9:949-956.

11. Paixao ES, Costa MCN, Teixeira MG, Harron K, Almeida MF, Barreto ML, Rodrigues LC. Symptomatic dengue during pregnancy and the risk of stillbirth: a matched case control study using routine data in Brazil (2006-2012). Lancet Infect Dis. **2017**;9:957-964.

12. Brazil, Ministry of Health. Manual de instruções para o preenchimento da declaração de nascido vivo. Fundação Nacional de Saúde Brasília; **2011**.

13. Oliveira, MM, Andrade SSCA, Dimech GS et al. Avaliação do Sistema de Informações sobre nascidos vivos. Brasil, 2006 a 2010. Epidemiol. E Serviços Saúde. **2015**;24:629–640.

14. Pedraza, D. F. Qualidade do Sistema de Informações sobre Nascidos Vivos (Sinasc): análise crítica da literatura. Cien Saude Colet. **2012**; 17:2729–2737.

15. Brazil, Ministry of Health. SINAN online- dengue and chikungunya instrucoes para preenchimento. Ficha de Investigacao. http://portalsinan.saude.gov.br/images/documentos/Agravos/Dengue/Instrucional_DENGUE_CHIK.pdf (Accessed May 4, 2017).

For peer review only

359 16. Villar J, Ismail LC, Victora CG, *et al.* International standards for newborn weight, length, and head circumference by gestational age and sex: the Newborn Cross-Sectional Study of the INTERGROWTH-21 st Project. The Lancet; **2014**; 384: 857–868.

362 17. Brazil, Ministry of Health. Dengue diagnostic e manejo clinic- adulto e crianca/Ministerio da Saude, Secretaria de Vigilancia em Saude, Diretoria Tecnica de gestao. 3 ed. Brasilia; **2016**.

365 18. Fellegi IP, Sunter AB. A theory for record linkage. J. Am. Stat. Assoc. **1969**; 64:1183–1210.

367 19. Paixao ES, Harron K, Andrade K, *et al.* Evaluation of record linkage of two large administrative databases in a middle income country: stillbirths and notifications of dengue during pregnancy in Brazil. BMC Med. Inform. Decis. Mak. **2017**; 1:108.

370 20. Williams R. Analyzing rare events with logistic regression. **2016**. https://www3.nd.edu/~rwilliam/stats3/RareEvents.pdf (Accessed May 4, 2017).

372 21. Doyle TJ, Goodin K, Hamilton JJ. Maternal and neonatal outcomes among pregnant women with 2009 pandemic influenza A (H1N1) illness in Florida, 2009-2010: a population-based cohort study. PLoS One. **2013**; 8: e79040.

375 22. Ogbuanu IU, Zeko S, Chu SY, et al. Maternal, fetal, and neonatal outcomes associated with measles during pregnancy: Namibia, 2009–2010. Clin. Infect. Dis. **2014**; 58:1086–1092.

377 23.Wedi CO, Kirtley S, Hopewell S, Corrigan R, Kennedy SH, Hemelaar J. et al. Perinatal outcomes associated with maternal HIV infection: a systematic review and meta-analysis. Lancet HIV. **2016**; 3:e33–e48.

For peer review only

24. Lee AC, Blencowe H, Cousens S, et al. National and regional estimates of term and preterm babies born small for gestational age in 18 low-income and middle-income countries in 2010. Lancet Glob Health. 2013; 1:e26-36.

25. Matijasevich A, Silveira MF, Matos ACG. Improved estimates of preterm birth prevalence in Brazil, 200-2011. Epidemiol. Serv. Saude. 2013; 22(4):557-564.

26. Brazil, Ministry of Health. Area Tecnica dengue, febre amarela e chikungunya. https://central3.to.gov.br/arquivo/249341/ (accessed July 10, 2017).

Funding

EP funded by National Council for Scientific and Technological Development (CNPq-Brazil); LCR is partially funded by the European Union's Horizon 2020 research and innovation program under Zika- PLAN grant agreement No. 734584; KH is funded by the Wellcome Trust (grant number 103975/Z/14/Z) However the funder of this study had no role in study design, data collection, data analysis, data interpretation, or writing of the report.

Data sharing

The data that support the findings of this study are available from Brazilian Ministry of Health but restrictions apply to the availability of these data, which were used under license for the current study, and so are not publicly available. Data are however available from the Brazilian Ministry of Health upon reasonable request.

Author Contributions Statement

ESP, KH and MBL carried out the analysis. ESP wrote the first draft of the article. LCR, MGT conceived the study. MCNC, MLB, OC, MAF contributed to the study design and interpretation. All authors revised the manuscript and approved the final version.

For peer review only

Conflict of interests

The authors declare that they have no competing interests.

For peer review only

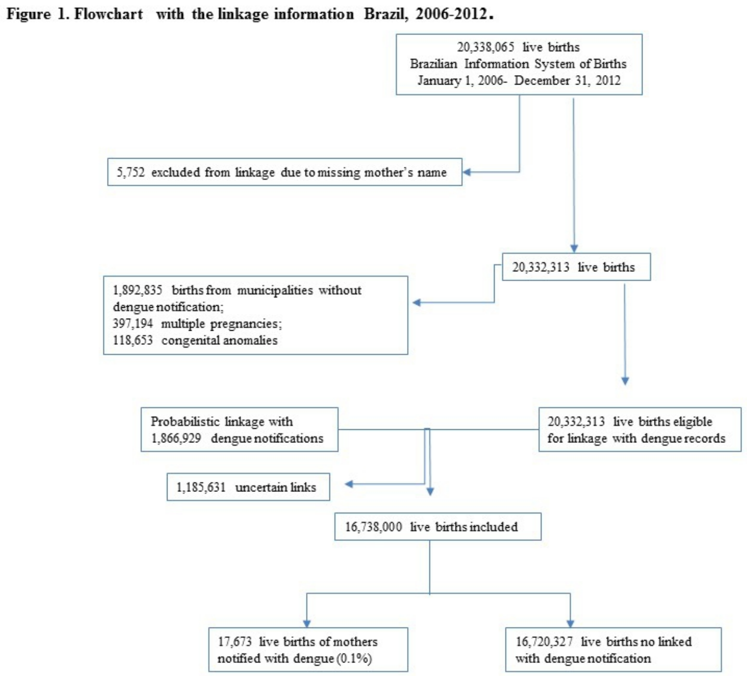

Figure 1. Flowchart with the linkage information Brazil, 2006-2012.

254x190mm (96 x 96 DPI)

STROBE Statement—Checklist of items that should be included in reports of ***cohort studies***

	Item No	Recommendation
**Title and abstract**	1	(*a*) Indicate the study's design with a commonly used term in the title or the abstract-page 1
		(*b*) Provide in the abstract an informative and balanced summary of what was done and what was found- page 2
**Introduction**		
Background/rationale	2	Explain the scientific background and rationale for the investigation being reported-page 4
Objectives	3	State specific objectives, including any prespecified hypotheses- page 4
**Methods**		
Study design	4	Present key elements of study design early in the paper- page 5 paragraph 89-91
Setting	5	Describe the setting, locations, and relevant dates, including periods of recruitment, exposure, follow-up, and data collection- page 5 this is a linkage study of pregnant women, therefore there specific details described during the methods section.
Participants	6	(*a*) Give the eligibility criteria, and the sources and methods of selection of participants. Describe methods of follow-up-page 7 paragraph 152
		(*b*) For matched studies, give matching criteria and number of exposed and unexposed- N/A
Variables	7	Clearly define all outcomes, exposures, predictors, potential confounders, and effect modifiers. Give diagnostic criteria, if applicable- page6-8
Data sources/ measurement	8*	For each variable of interest, give sources of data and details of methods of assessment (measurement). Describe comparability of assessment methods if there is more than one group-page 6-8 and table 1
Bias	9	Describe any efforts to address potential sources of bias- page 8
Study size	10	Explain how the study size was arrived at- figure 1
Quantitative variables	11	Explain how quantitative variables were handled in the analyses. If applicable, describe which groupings were chosen and why- n/a
Statistical methods	12	(*a*) Describe all statistical methods, including those used to control for confounding-page 8
		(*b*) Describe any methods used to examine subgroups and interactions- page 8
		(*c*) Explain how missing data were addressed- page 8
		(*d*) If applicable, explain how loss to follow-up was addressed N/A
		(*e*) Describe any sensitivity analyses- page 8
**Results**		
Participants	13*	(a) Report numbers of individuals at each stage of study—eg numbers potentially eligible, examined for eligibility, confirmed eligible, included in the study, completing follow-up, and analysed- figure 1and page
		(b) Give reasons for non-participation at each stage-figure 1
		(c) Consider use of a flow diagram-figure 1 and page 9
Descriptive data	14*	(a) Give characteristics of study participants (eg demographic, clinical, social) and information on exposures and potential confounders-table 1page 9
		(b) Indicate number of participants with missing data for each variable of interest-table 1
		(c) Summarise follow-up time (eg, average and total amount)- n/a
Outcome data	15*	Report numbers of outcome events or summary measures over time- table 1 page 9-10

For peer review only - http://bmjopen.bmj.com/site/about/guidelines.xhtml
For peer review only

Main results	16	(*a*) Give unadjusted estimates and, if applicable, confounder-adjusted estimates and their precision (eg, 95% confidence interval). Make clear which confounders were adjusted for and why they were included- table 2-4
		(*b*) Report category boundaries when continuous variables were categorized-methods section page 8
		(*c*) If relevant, consider translating estimates of relative risk into absolute risk for a meaningful time period-n/a
Other analyses	17	Report other analyses done—eg analyses of subgroups and interactions, and sensitivity analyses- page 8
**Discussion**		
Key results	18	Summarise key results with reference to study objectives-page 13
Limitations	19	Discuss limitations of the study, taking into account sources of potential bias or imprecision. Discuss both direction and magnitude of any potential bias page 15
Interpretation	20	Give a cautious overall interpretation of results considering objectives, limitations, multiplicity of analyses, results from similar studies, and other relevant evidence-page 15
Generalisability	21	Discuss the generalisability (external validity) of the study results- page 15
**Other information**		
Funding	22	Give the source of funding and the role of the funders for the present study and, if applicable, for the original study on which the present article is based-page 19

*Give information separately for exposed and unexposed groups.

Note: An Explanation and Elaboration article discusses each checklist item and gives methodological background and published examples of transparent reporting. The STROBE checklist is best used in conjunction with this article (freely available on the Web sites of PLoS Medicine at http://www.plosmedicine.org/, Annals of Internal Medicine at http://www.annals.org/, and Epidemiology at http://www.epidem.com/). Information on the STROBE Initiative is available at http://www.strobe-statement.org.

For peer review only

BMJ Open

Dengue during pregnancy and live birth outcomes: a cohort of linked data from Brazil

Journal:	*BMJ Open*
Manuscript ID	bmjopen-2018-023529.R1
Article Type:	Research
Date Submitted by the Author:	23-Jan-2019

Complete List of Authors:	Paixão, Enny; London School of Hygiene and Tropical Medicine Faculty of Epidemiology and Population Health, IDE<br>Campbell, Oona; London School of Hygiene and Tropical Medicine Faculty of Epidemiology and Population Health, IDE<br>Teixeira, Maria; Universidade Federal da Bahia<br>Costa, Maria Conceição; Federal University of Bahia, Instituto de Saúde Coletiva<br>Harron, Katie; London School of Hygiene and Tropical Medicine, Department of Health Services Research and Policy<br>Barreto, Mauricio; Instituto de Saude Coletiva/UFBa,<br>Leal, Maira; Universidade Federal da Bahia<br>Almeida, Marcia; Universidade de São Paulo, School of Public Health<br>Rodrigues, Laura; London School of Hygiene and Tropical Medicine,

**Primary Subject Heading**:	Epidemiology
Secondary Subject Heading:	Epidemiology, Infectious diseases
Keywords:	Dengue, pregnancy, birth outcomes

SCHOLARONE™ Manuscripts

Dengue during pregnancy and live birth outcomes: a cohort of linked data from Brazil

Running title: Dengue and adverse birth outcomes

Enny S Paixão*, MSc[abc]

Oona M.R.Campbell, PhD[a]

Maria Glória Teixeira, PhD[bc]

Maria da Conceição N. Costa, PhD[b]

Katie Harron, PhD[a]

Mauricio L. Barreto,PhD[bc]

Maira B. Leal[b]

Marcia Furquim de AlmeidaPhD[d]

Laura C. Rodrigues, PhD[ac]

For peer review only

* Corresponding author email: npaixaoenfo@yahoo.com.br; Phone 44 02079588171

a London School of Hygiene and Tropical Medicine. Keppel St, Bloomsbury, London WC1E 7HT, United Kingdom

b Institutode Saúde Coletiva. Rua Basílio da Gama, s/n.Canela. CEP 40110040. Salvador, Bahia, Brasil.

c Center of Data and Knowledge Integration for Health (CIDACS), Instituto Gonçalo Moniz, Fundação Oswaldo Cruz, CEP 41745-715 Salvador-Bahia, Brazil

d Universidade de São Paulo.Av. Dr. Arnaldo, 715 Cerqueira Cesar, 01246904 - São Paulo, SP – Brasild

Abstract

Objectives: Dengue is the most common viral mosquito-borne disease, and women of reproductive age who live in or travel to endemic areas are at risk. Little is known about the effects of dengue during pregnancy on birth outcomes. The objective of this study is to examine the effect of maternal dengue severity on live birth outcomes.

Design and setting: We conducted a population-based cohort study using routinely-collected Brazilian data from 2006-2012.

Participating: We linked birth registration records and dengue registration records to identify women with and without dengue during pregnancy. Using multinomial logistic regression and Firth method, we estimated risk and odds ratios (OR) for preterm birth (<37 weeks gestation), low birth weight (<2500g) and small for gestational age (<10th centile). We also investigated the effect of time between the onset of the disease and each outcome.

Results: We included 16,738,000 live births. Dengue haemorrhagic fever was associated with preterm birth (OR=2.4; 95%CI 1.3-4.4) and low birth weight (OR=2.1;95%CI 1.1-4.0) but there was no evidence of effect for small for gestational age (OR=2.1;95% CI 0.4-12.2). The magnitude of the effects was higher in the acute disease period.

Conclusion: This study showed an increased risk of adverse birth outcomes in women with severe dengue during pregnancy. Medical intervention to mitigate maternal risk during severe acute dengue episodes may improve outcomes for infants born to exposed mothers.

Strengths and limitations of this study

- This is the first study to investigate the risk of small for gestational age infants born to women with dengue during pregnancy using a population-based approach with a sufficiently large sample size and controlling for confounders.

For peer review only

- Due to sample size we were able to investigate rare exposure (such as dengue haemorrhagic fever)
- This study relies on the use of secondary data, and therefore identification of the outcome and the exposure is susceptible to misclassification.
- The linkage process was rigorously validated and although we were unable to capture all cases, it is unlikely that the linkage process introduced bias, since linkage errors were not associated with any of our variables of interest.

For peer review only

Introduction

Dengue is the most common viral vector-borne disease worldwide, with an estimated 390 million people infected each year, 96 million of whom develop clinical symptoms. It is endemic in over 100 countries (mostly in South and Central American and Southeast Asia), and is spreading to new areas; according to the World Health Organization (WHO), approximately half of the world's population is at risk [1]. The effects of dengue during pregnancy are unclear, and there is a lack of evidence to inform women of reproductive age who live in or travel to endemic areas and who are at risk of dengue. The majority of studies published to date are cases series[2]; the seven studies published before 2016 with comparison groups show conflicting results [3456789] and a meta-analysis published in 2016 found dengue during pregnancy was only associated with low birth weight and preterm birth in symptomatic women[2]. In 2017, a large Brazilian study found that notification of mild dengue during pregnancy was not associated with higher rates of preterm birth or lower birth weight in neonates compared to rates in a random sample of all neonates. However, this study did not investigate the effects of dengue by severity or date of disease onset[10].

Maternal dengue, and specifically severe dengue, has been associated with fetal deaths,[11] but the effects on those who survive has not been investigated in population-based study. We analysed a large, population-based cohort to investigate the association between symptomatic dengue notified during pregnancy and adverse birth outcomes, and to examine the effect of maternal dengue severity.

For peer review only

Methods

We performed a population-based cohort study by linking routine records of live births with records of women notified and confirmed with dengue disease, in Brazil from January 1, 2006 until December 31, 2012. The methods described in this paper are similar to those used in a previously published paper about the effects of dengue on maternal deaths[12].

Data sources

We used the following data from two Brazilian databases.

1) The Live Births Information System (Sistema de Informação sobre Nascimentos; SINASC), which records live births in Brazil; these data are derived from the birth registration, a legal document completed by the health worker who attended the birth. It includes information on the mother (name, place of residence, age, marital status, education); the pregnancy (length of gestation, mode of delivery); and the neonate (birth weight, presence of congenital anomalies) [13]. Data completeness is very high, with 97% of Brazilian births registered,[14] and most variables were >90% complete[15].

2) Notifiable Diseases Information System (Sistema de Informação de Agravos de Notificação; SINAN), which records notifiable diseases. Dengue notifications include information on the individual (name, place of residence, age, sex, and years of education), symptoms of the disease, laboratory tests, and disease severity, assessed clinically and by laboratory according with the Brazilian dengue manual [16]. These data are reasonably complete on the variables used for linkage: <0.05% records were excluded because of a missing name. Around 5% of dengue cases did not have a final classification of severity. Laboratory confirmation was not required for notification or confirmation of dengue. During the study period (2006-2012), dengue was the main (and sometimes the only) vector-borne disease circulating in Brazil, as yellow fever

For peer review only

and malaria occurred in restricted areas, and Zika and Chikungunya did not begin to circulate until 2014.

Procedures

SINASC defines a live birth as the product of conception that, independent of the duration of pregnancy, breathes or shows any other signs of life (such as a heartbeat, umbilical cord pulsation, or definite movement of voluntary muscles), after the separation from the mother's body [13]. We excluded live birth records from twin or other multiple pregnancies, congenital anomalies, and records from municipalities without dengue notifications (Figure 1).

Outcomes

We assessed three birth outcomes: preterm birth (<37 weeks); low birth weight (<2500g) and small for gestational age, calculated according to the Intergrowth scale [17] using birth weight and gestational age in weeks at birth. This scale is sex specific, and those babies with a birth weight less than the 10th centile were considered small for gestational age. The data to calculate small for gestational age were available only for 2011 and 2012 (prior to this, gestational age was categorized).

Exposure

We defined our exposure as confirmed cases of dengue notified to SINAN during a pregnancy that resulted in a live birth. In Brazil, confirmation of dengue is based either on clinical and epidemiological criteria (presence of clinical symptoms of dengue in the same area and time as other confirmed cases of dengue), or on clinical and laboratory criteria (the presence of clinical symptoms and a positive test for one of: a) IgM detection by ELISA, b) viral RNA detection via PCR, c) NS1 viral antigen detection, or d) positive viral culture). We refer to laboratory confirmed cases as “dengue during pregnancy, laboratory confirmed”. Dengue notifications do not always state whether the woman notified was pregnant – there is a field for this variable, but it is largely missing. We therefore defined maternal dengue cases by linking SINAN

For peer review only

confirmed dengue cases with live birth registrations within the nine months following disease notification. To categorise disease severity, we used the clinical classification valid in Brazil in the period in which data were collected, which classified dengue into three clinical categories: a) “dengue fever”, b) “complicated dengue”, and c) “dengue haemorrhagic fever/ dengue shock syndrome”. Dengue fever is a self-limiting disease, characterized as fever, with a severe headache, pain behind the eyes, muscle and joint pain, and rash; Complicated dengue is a probable case of dengue characterized by one of the following: severe changes in the nervous system, cardiorespiratory dysfunction; insufficient hepatic function; gastrointestinal bleeding, cavity spills, or thrombocytopenia equal or less than 50,000/mm^3, or leucometry less than 1000/mm;[18] Dengue haemorrhagic fever follows the WHO criteria and is characterized by fever, haemorrhagic evidence, thrombocytopenia and evidence of plasma leakage. Complicated dengue is a Brazilian definition for severe cases of dengue that do not meet WHO criteria for dengue haemorrhagic fever and cannot be classified as mild self-limited disease (Dengue fever) due to their severity.

Linkage process

We linked records of women with confirmed dengue (SINAN) with records of live births (SINASC) up to nine months after the SINAN notification, to identify those mothers who had dengue during pregnancy. Since there was no unique identifier for linkage between the two data sources, we relied on a set of identifiers available with good data quality in both datasets: name, age or date of birth, and the place of residence of the mother at time of delivery / time of notification. We excluded records with missing or implausible names (e.g. the name of a hospital as the name of the mother)(Figure 1).

For peer review only

We used probabilistic linkage, which generates match weights that represent the likelihood that two records belong to the same individual, based on the similarity between records from different datasets. This linkage method takes into account errors and missing values in identifiers, and is useful when there is no unique identifier. Match weight calculations used the Fellegi-Sunter method.[19] For each record pair we calculated a probabilistic match weight based on two conditional probabilities: m-probability, P (agreement|match) and u-probability, P (agreement|non-match). Match weights were calculated by summing the log of the ratio of m-probabilities and u-probabilities across different identifiers. The algorithm was implemented in Stata 14.1 and R 3.4.1.

Linkage followed the same procedure as previously described for linking stillbirth records and the dengue database in Brazil, in which we demonstrated a sensitivity (the proportion of true links captured) of 62% and a positive predictive value (the proportion of true matches among linked records) of 95%.[20] To estimate parameters for linkage weights and to validate the quality of the linkage, we created a gold-standard dataset based on data from two states for 2010 Ceará and Espirito Santo). These states were chosen as they included a manageable number of records with which to conduct manual review different, and represented a wide range of social economic status; 2010 was chosen since this was dengue epidemic year. Record pairs were ordered by match weight and manually reviewed to establish true matches (records belonging to the same mother) and to identify any erroneous links (false matches). Live birth records linked to confirmed dengue notifications with high certainty were classified as dengue in pregnancy cases, and birth records we were confident had no link were classified as non-dengue cases. We excluded any records for which there was an uncertain link (where we could not establish whether a woman had dengue) to avoid misclassification (Figure 1). Following linkage, the data were de-identified prior to analysis.

For peer review only

Statistical analysis

We estimated the crude and adjusted risk ratios (RR) and confidence intervals (CI) from multinomial logistic regression. We compared live births from women without dengue during pregnancy (our reference group) with live births from women with notified dengue during pregnancy. We controlled for maternal age (categorized as <=20; between 20-35 and >=35 years old), education (less than 3 years; between 4-7 years and more than 8 years) and marital status (single/ widow /divorced or married/stable union), as these variables were also available in the data. To assess the sensitivity of results to the validity of clinical/epidemiologic diagnosis, we repeated the analyses using laboratory confirmed dengue cases only.

In general, dengue is an acute disease with rapid recovery. However, we investigated the effect of time between disease onset and live birth outcomes. The time between disease onset and birth outcome was calculated using the date of the disease onset (information available in SINAN) and the date when the outcome occurred (date of live birth); we categorized this difference as being less than or equal to ten days or greater than 10 days.

We also evaluated the association between dengue and birth outcomes according to severity of disease. The exposure was classified as mild dengue, dengue with complications and dengue haemorrhagic fever. For this analysis, we calculated the odds ratio (OR) using the Firth method (to reduce the small sample bias in maximum likelihood estimation) as we were analysing rare events, and controlled for maternal age, education and marital status [21].

Patient and Public Involvement

There was no patients involved in the research. The data used are administrative routinely collected data.

For peer review only

Ethics Statements

Ethical approval was obtained from The Federal University of Bahia, Salvador, Brazil (CAAE: 26797814.7.0000.5030) and from The London School of Hygiene and Tropical Medicine (Ethics Ref:10269).

Results

A total of 20,333,482 live births were recorded in SINASC between 2006-2012. After exclusions, 16,738,000 live births were included in the study, and 17,673 (0.1%) of their mothers were linked with a dengue notification record (Figure 1).

The cohort characteristics by dengue status are described in Table 1. Compared to pregnant women without dengue, pregnant women with dengue were more likely to have more years of formal education and to have delivered by caesarean-section.

Table 1. Maternal characteristics, delivery details, and birth outcomes in relation to dengue status, Brazil, 2006-2012

Characteristics	Notified with confirmed dengue in pregnancy<br>n (%)	Without dengue notification<br>n (%)
**Age of the mother**		
< 20	4,499 (25.5)	4,384,159 (26.2)
20-35	11,981 (67.8)	11,042,342 (66.0)
>35	1,193 (6.7)	1,293,171 (7.8)
Missing	-	655 (0.0)
**Maternal education**		
Less than 3 years	1,065 (6.1)	1,358,815 (8.3)
4-7 years	4,661 (27.0)	4,592,979 (28.1)
More than 8 years	11,578 (66.9)	10,399,703 (63.6)
Missing	369 (2.1)	368,830 (2.2)
**Marital status**		
Single/Widow/Divorced	11,518 (66.1)	9,660,306 (58.7)
Married /Union	5,899 (33.9)	6,797,658 (41.3)

For peer review only

Missing	256 (1.4)	262,363 (1.6)
**Numbers of pre-natal visits**		
Inadequate (less than 7 times)	6,991 (39.9)	6,817,679 (41.2)
Adequate (7 or more times)	10,515 (60.1)	9,712,338 (58.8)
Missing	167 (0.9)	190,310 (1.1)
**Delivery**		
Vaginal	8,332 (47.2)	8,383,714 (50.2)
C-section	9,320 (52.8)	8,307,072 (49.8)
Missing	21 (0.1)	29,541 (0.2)
**Gestational age in the delivery**		
Less than 22 weeks	17 (0.1)	8,844 (0.0)
22-27 weeks	68 (0.4)	58,760 (0.4)
28-31 weeks	147 (0.8)	112,407 (0.7)
32-36 weeks	1,157 (6.7)	1,011,633 (6.1)
Total preterm birth	1,389 (8.0)	1191644 (7.2)
More than 37 weeks	15,994 (92.0)	15,215,440 (92.8)
Missing	290 (1.6)	313,243 (1.9)
**Birth weight**		
>=3,000	12,171 (69.1)	11,659,487 (69.9)
3,000-2,500	3,973(22.5)	3,816,201 (22.9)
Total low birth weight	1,484 (8.4)	15,475,688 (7,2)
1,500-2,500	1,258 (7.1)	1,026,248 (6.1)
<1,500	226 (1.3)	178,643 (1.1)
Missing	38 (0.2)	39,748 (0.2)
**Small for gestational age (10th centile)#**		
Normal	3,681 (91.7)	3,289,125 (92.0)
Small	331 (8.2)	287,173 (8.0)
Data not collected	13,661 (77.3)	13,144,029 (78.6)

*% of each category without the missing value
# data available only in 2011 and 2012

Maternal dengue was associated with a slight increase in the risk of preterm birth (from 7.3% to 7.9%, RR 1.1; 95% CI 1.0-1.2) and low birth weight (from 7.2% to 8.4%, RR 1.2; 95% CI 1.1-1.2), although the confidence interval was borderline. There was no association between maternal dengue and being small for gestational age (Table 2). Restricting analyses to the 33%, (5,755/17,388) of dengue cases that were laboratory confirmed did not change the magnitude of the associations (Table 2).

For peer review only

Table 2. Number of cases notified with dengue during pregnancy (N), crude and adjusted risk ratio for the association among dengue during pregnancy and preterm birth, low birth weight, small for gestational age. Brazil, 2006-2012.

	All dengue cases			Lab confirmed dengue cases		
	N	Crude Risk Ratio (95 % Confidence Interval)	Adjusted Risk Ratio (95 % Confidence Interval)		Crude Risk Ratio (95% Confidence Interval)	Adjusted Risk Ratio (95% confidence Interval)
**Preterm birth N=1,193,033**						
Overall (<37 weeks)	1,389	1.1 (1.0-1.2)	1.1 (1.0-1.2)	439	1.1 (1.0-1.2)	1.1 (1.0-1.2)
Less than 28 weeks		1.1 (1.0-1.1)	1.1 (1.0-1.2)		1.0 (0.9-1.2)	1.0 (0.9-1.2)
28-32 weeks		1.2 (1.1-1.5)	1.2 (1.1-1.4)		1.2 (0.9-1.6)	1.2 (0.9-1.6)
32-36 weeks		1.2 (1.0-1.5)	1.1 (0.9-1.4)		1.1 (0.7-1.6)	1.1 (0.7-1.6)
**Low birth weight N=1,206,375**						
Overall (<2500g)	1484	1.2 (1.1-1.2)	1.2 (1.1-1.2)	475	1.2 (1.0-1.3)	1.2 (1.1-1.3)
1500g-2499g		1.2 (1.1-1.2)	1.2 (1.1-1.2)		1.2 (1.0-1.3)	1.2 (1.1-1.3)
Less than 1500g		1.2 (1.1-1.4)	1.2 (1.0-1.3)		1.1 (0.9-1.4)	1.1 (0.9-1.4)
**Small for gestational age N=287,504**						
<10th centile	331	1.0 (0.9-1.1)	1.0 (0.9-1.1)	68	0.9 (0.7-1.1)	0.9 (0.7-1.2)

Adjusted for maternal age, education, marital status

Table 3 shows the dose-response effect of the severity of dengue on preterm birth, low birth weight and small for gestational age. Dengue haemorrhagic fever in pregnancy was associated with a doubling of the risk of preterm birth, from 1,191,644/16,407,084 (7.3%) to 12/79 (15.2%); OR 2.4 (95% CI 1.3-4.4) and an increase in the odds of low birth weight, from 1,204,891/16,680,579 (7.2%) to 11/79 (13.9%); OR 2.1 (95% CI 1.1-4.0). We found no evidence for an effect of dengue on small for gestational age, even among pregnant women who developed haemorrhagic disease (287,173/3,576,298 (8.0%) versus 1/9 (11.1%); OR 2.5 (95% CI 0.4-12.2).

Table 3. Number of cases with dengue during pregnancy (N) and Odds Ratio (OR) for the association Dengue during pregnancy by severity of disease and adverse birth outcomes (preterm birth, low birth weight, small for gestational). Brazil, 2006-2012.

For peer review only

Outcome	Dengue fever	Complicated dengue	Haemorrhagic fever
**Preterm birth (<37 weeks) N=1,192,920**			
N (%)	1,234	30	12
Crude OR (95% CI)	1.1 (1.0-1.1)	1.4 (0.9-1.9)	2.4 (1.3-4.3)
Adjusted OR (95% CI)	1.1 (1.0-1.1)	1.4 (0.9-2.0)	2.4 (1.3-4.4)
**Low birth weight (<2500g) N=1,206,265**			
N	1,327	36	11
Crude OR (95% CI)	1.1 (1.1-1.2)	1.6 (1.2-2.3)	2.1 (1.1-4.0)
Adjusted OR (95% CI)	1.1 (1.1-1.2)	1. 6 (1.1-2.3)	2.1 (1.1-4.0)
**Small for gestational age (10th centile) N=287,474**			
N	294	6	1
Crude OR (95% CI)	1.0 (0.9-1.1)	2.2 (0.9-5.1)	2.0 (0.3-11.4)
Adjusted OR (95% CI)	1.0 (0.9-1.1)	2.3 (1.0-5.3)	2.1 (0.4-12.2)

Adjusted for maternal age, education, marital status

There was some evidence that the risk of dengue in pregnancy on birth outcomes depended on the time between dengue onset and the date of live birth: the magnitude of the effect of dengue on adverse birth outcomes was higher during the acute disease period, with some residual effects remaining after the first 10 days for preterm birth and low birth weight (Table 4).

For peer review only

Table 4.Number of cases with dengue during pregnancy (N) and risk Ratio (RR) for the association Dengue during pregnancy by timing of disease and adverse birth outcomes (preterm birth, low birth weight, small for gestational). Brazil, 2006-2012.

**Outcome**	**Outcomes within 10 days of disease onset (95 % Confidence Interval)**	**Outcomes after 10 days from disease onset (95 % Confidence Interval)**
**Preterm birth**		
**N=1,191,719**		
N	75	1,314
Crude RR (95% CI)	2.1 (1.7-2.7)	1.1 (1.0-1.1)
Adjusted RR (95% CI)	2.0 (1.6-2.6)	1.1 (1.0-1.1)
**Low birth weight**		
**N= 1,206,375**		
N	74	1410
Crude RR (95% CI)	2.1 (1.6-2.6)	1.1 (1.1-1.2)
Adjusted RR (95% CI)	2.0 (1.6-2.6)	1.1 (1.1-1.2)
**Small for gestational age (<10th centile)**		
**N=287,173**		
N	4	83
Crude RR (95% CI)	0.5 (0.2-1.5)	1.0 (0.9-1.1)
Adjusted RR (95% CI)	0.5 (0.2-1.5)	1.0 (0.9-1.1)

Adjusted for maternal age, education, marital status

Discussion

Our study demonstrates that dengue haemorrhagic fever in pregnancy is associated with a doubling of the risk of preterm birth (OR: 2.4) and of low birth weight (OR: 2.1), and that mild dengue fever is associated with an increase of 10 to 20 percent in the risk of preterm birth and

For peer review only

low birth weight respectively. We found no evidence for an increased risk of small for gestational age for either severe or mild disease. The association between dengue and birth outcomes was strongest during the acute disease phase, within the first ten days of disease onset.

Our results are consistent those with Nascimento, et al.[10] who showed that symptomatic dengue infection did not greatly affect the risk of low birthweight (1.00 95% CI 0.85-1.17) or preterm birth (OR: 0.98 95% CI 0.83-1.16) when compared to a random sample of live births. However, we have previously shown that mild dengue is associated with a doubling of the risk of stillbirth (and that severe cases are associated with a 5-fold increase in risk). By considering only live birth outcomes and excluding births with congenital anomalies (<1% of the sample) in this study, we may be underestimating the effect of dengue during pregnancy. Our findings are also consistent with a meta-analyses published in 2016,[2] which found an association between symptomatic dengue and preterm birth and low birth weight within in a hospital based population that sought care, and was likely to include only severe dengue cases. In our study, the association between maternal dengue and birth outcomes was strongest for women with dengue haemorrhagic fever in pregnancy.

There is evidence that different infectious diseases in pregnancy can lead to adverse outcomes. The evidence is still fragmented, and there is no consistent effect, which appears to vary according to the pathogen involved, and the timing and severity of maternal disease.For instance, studies comparing pregnant women with infection to pregnant women without, found a) severe influenza increased the risk of preterm birth from 2.4 to 4 times, whereas studies based on mild range of illness did not find an effect; [22] b) measles and HIV infection increased the risk of low birth weight by 3.5 and 1.6 times, respectively. [2324]

One mechanism by which dengue during pregnancy affects preterm birth and low birth weight that is consistent with our results, is through maternal illness, rather than a direct effect on the

For peer review only

fetus. Since we did not observe an effect on small for gestational age, the effect of dengue in pregnancy on birth weight may have occurred via prematurity of the newborn. This assumption is supported by the higher risk of preterm births observed among mothers within less than 10 days after the onset of the disease and among those with complicated disease and haemorrhagic dengue. The higher risk of preterm birth could be due to the early onset of labour delivery or by early delivery due to medical interventions, such as caesarean section, required because of concern about risk to the mother. However, data on small for gestational age was only available for 2011-2012, which may have affected our ability to observe an effect. Furthermore, analysis according to mode of delivery is complicated due to high rates of caesarean section in Brazil, many of which are not clinically indicated.

Limitations of this study include the linkage process and use of secondary data. Firstly, outcome and exposure identification is susceptible to misclassification using secondary sources. Preterm birth and small for gestational age are under-ascertained in routinely-collected data from low and middle income countries. However as this is likely to have affected both those exposed and not exposed to dengue in a similar way, it is unlikely that results of this study would have been biased.[25, 26] Regarding the dengue diagnosis, it is usual practice in outbreaks/ epidemics to test until the origin of the outbreak is clearly established, and after that only test when there is a clinical indication. The proportion of laboratory confirmed cases among general population in Brazil is around 30%, [27] and therefore misclassification based on the lack of laboratory confirmation is possible. However, our sensitivity analysis demonstrated similar results in subsets of the data with and without laboratory confirmation of dengue. Secondly, our linkage was rigorously validated in a previous study.[19] Despite the low sensitivity (that kept us from making statements about the prevalence of dengue during pregnancy) it is unlikely that the linkage process introduced bias, since linkage errors occurred

For peer review only

randomly within the data and were not associated with our variables of interest. Finally, although we adjusted for confounders, other possible unknown confounders, such as maternal co-morbidities or quality or type of obstetric care, may have contributed to the association between severe dengue and adverse birth outcomes.

As dengue prevention is not straightforward and mosquito control has failed in Brazil, our findings highlight the crucial role of the clinical management of dengue in pregnant women. Doctors and medical staff in general attending to pregnant women with dengue should closely observe and monitor the patients to be able to intervene timely and avoid adverse outcomes for mothers and babies, especially during the acute phase. We recommend careful observation and notation of dengue in antenatal records and further research into the association between dengue and adverse pregnancy outcomes.

In summary, this study demonstrates a doubling in the risk of preterm birth and low birth weight associated with dengue haemorrhagic fever in pregnancy, and increases of 10-20% in women with mild dengue. One contributing factor to adverse birth outcomes may be medical interventions triggered to mitigate mothers risk associated with dengue haemorrhagic fever. We recommend further research in different settings to confirm our results, and additional studies of adverse birth outcomes for other vector-borne diseases.

For peer review only

References

1. World Health Organization. Dengue and severe dengue. WHO Fact Sheet No117 Updated. April. 2017. http://www.who.int/mediacentre/factsheets/fs117/en/ (Accessed May 4, 2017).

2. Paixao ES, Teixeira, MG, Costa, MCN, Rodrigues LC. Dengue during pregnancy and adverse fetal outcomes: a systematic review and meta-analysis. Lancet Infect. Dis.**2016**;16:857-865.

3. Friedman EE, Dallah F, Harville EW et al. Symptomatic dengue infection during pregnancy and infant outcomes: a retrospective cohort study. PLoS Negl Trop Dis **2014**; **8**: e3226.

4. Tan PC, Rajasingam G, Devi S, Omar SZ. Dengue infection in pregnancy: prevalence, vertical transmission, and pregnancy outcome. Obstet Gynecol **2008**; 111: 1111–17.

5. Restrepo BN, Isaza DM, Salazar CL, et al. Dengue y embarazoen Antioquia, Colombia. Revista Facultad Nacional de Salud Pública **2004**; 22: 7–14.

6. Alvarenga CF, Silami VG, Brasil P, Boechat MEH, Coelho J, Nogueira RMR. Dengue during pregnancy: a study of thirteen cases. Am J Infect Dis **2009**; 5: 6.

7. Angarita LCR, Angarita SV, Correa M, Odreman MI. Transmisión perinatal del virus dengue en el binomiomadre-hijo. Arch Venez Pueric Pediatr **2003**; 76: 99–104.

8. Barroso LR, Betancourt ID, Saeta YF, Navarro MM, Guerra GD. Repercusión del dengue serotipo 3 sobre el embarazo y producto de la concepción. Rev Cuba Obstet Ginecol **2010**; 36: 42–50

9. Leite RC, Souza AI, Castanha PM, et al. Dengue infection in pregnancy and transplacental transfer of anti-dengue antibodies in Northeast, Brazil. J Clin Virol **2014**; 60: 16–21.

10. Nascimento LB, Siqueira CM, Coelho GE, Siqueira JB. Symptomatic dengue infection during pregnancy and livebirth outcomes in Brazil, 2007–13: a retrospective observational cohort study. Lancet Infect Dis. **2017**;9:949-956.

For peer review only

11. Paixao ES, Costa MCN, Teixeira MG, Harron K, Almeida MF, Barreto ML, Rodrigues LC. Symptomatic dengue during pregnancy and the risk of stillbirth: a matched case control study using routine data in Brazil (2006-2012). Lancet Infect Dis. **2017**;9:957-964.

12. Paixao ES, Harron K, Campbell O, Teixeira MG, Costa MDCN, Barreto ML, Rodrigues LC. Dengue in pregnancy and maternal mortality: a cohort using routine data. Sci Rep. 2018 Jul 2;8(1):9938. doi: 10.1038/s41598-018-28387-w.

13. Brazil, Ministry of Health. Manual de instruções para o preenchimento da declaração de nascido vivo. Fundação Nacional de Saúde Brasília; **2011**.

14. Oliveira, MM, Andrade SSCA, Dimech GS et al. Avaliação do Sistema de Informações sobre nascidos vivos. Brasil, 2006 a 2010. Epidemiol. E Serviços Saúde. **2015**;24:629–640.

15. Pedraza, D. F. Qualidade do Sistema de Informações sobre Nascidos Vivos (Sinasc): análise crítica da literatura. Cien Saude Colet.**2012**; 17:2729–2737.

16. Brazil, Ministry of Health. SINAN online- dengue and chikungunya instrucoes para preenchimento. Ficha de Investigacao. http://portalsinan.saude.gov.br/images/documentos/Agravos/Dengue/Instrucional_DENGUE_CHIK.pdf (Accessed May 4, 2017).

17. Villar J, Ismail LC, Victora CG, *et al.* International standards for newborn weight, length, and head circumference by gestational age and sex: the Newborn Cross-Sectional Study of the INTERGROWTH-21 st Project. The Lancet; **2014**;384: 857–868.

For peer review only

18. Brazil, Ministry of Health. Dengue diagnostic e manejo clinic- adulto e crianca/Ministerio da Saude, Secretaria de Vigilancia em Saude, Diretoria Tecnica de gestao. 3 ed. Brasilia; **2016**.

19. Fellegi IP, Sunter AB. A theory for record linkage. J. Am. Stat. Assoc.**1969**; 64:1183–1210.

20. Paixao ES, Harron K, Andrade K, *et al.* Evaluation of record linkage of two large administrative databases in a middle income country: stillbirths and notifications of dengue during pregnancy in Brazil. BMC Med. Inform. Decis. Mak. **2017**; 1:108.

21. Williams R. Analyzing rare events with logistic regression. **2016**. https://www3.nd.edu/~rwilliam/stats3/RareEvents.pdf (Accessed May 4, 2017).

22. Doyle TJ, Goodin K, Hamilton JJ. Maternal and neonatal outcomes among pregnant women with 2009 pandemic influenza A (H1N1) illness in Florida, 2009-2010: a population-based cohort study. PLoS One. **2013**;8: e79040.

23. Ogbuanu IU, Zeko S, Chu SY, et al. Maternal, fetal, and neonatal outcomes associated with measles during pregnancy: Namibia, 2009–2010. Clin. Infect. Dis.**2014**; 58:1086–1092.

24. Wedi CO, Kirtley S, Hopewell S, Corrigan R, Kennedy SH, Hemelaar J. et al. Perinatal outcomes associated with maternal HIV infection: a systematic review and meta-analysis. Lancet HIV. **2016**;3:e33–e48.

25. Lee AC, Blencowe H, Cousens S, et al. National and regional estimates of term and preterm babies born small for gestational age in 18 low-income and middle-income countries in 2010. Lancet Glob Health. 2013; 1:e26-36.

For peer review only

26. Matijasevich A, Silveira MF, Matos ACG. Improved estimates of preterm birth prevalence in Brazil, 200-2011. Epidemiol. Serv. Saude. 2013; 22(4):557-564.

27. Brazil, Ministry of Health. Area Tecnica dengue, febre amarela e chikungunya. https://central3.to.gov.br/arquivo/249341/ (accessed July 10, 2017).

Funding

EP funded by National Council for Scientific and Technological Development (CNPq-Brazil); LCR is partially funded by the European Union's Horizon 2020 research and innovation program under Zika- PLAN grant agreement No. 734584; KH is funded by the Wellcome Trust (grant number 103975/Z/14/Z) However the funder of this study had no role in study design, data collection, data analysis, data interpretation, or writing of the report.

Data sharing

The data used in this study is identified, therefore confidential, because they include patient personal information that can be traced back to individual. They are obtainable in the Brazilian Ministry of Health but restrictions apply to the availability of these data, which were used under license for the current study, and so are not publicly accessible. Data are however available from the Brazilian Ministry of Health upon reasonable request for researchers who meet the criteria for access to confidential data, approved by the Ethics Committee. Email of a contact point in the Department of Health Information Ministry of Health Brazil:datasus@saude.gov.br.

Author Contributions Statement

ESP, KH and MBL carried out the analysis. ESP wrote the first draft of the article. LCR, MGT conceived the study. MCNC, MLB, OC, MAF contributed to the study design and interpretation. All authors revised the manuscript and approved the final version.

Conflict of interests

For peer review only

The authors declare that they have no competing interests.

Figure 1. Flowchart with the linkage information Brazil, 2006-2012

For peer review only

Figure 1. Flowchart with the linkage information Brazil, 2006-2012.

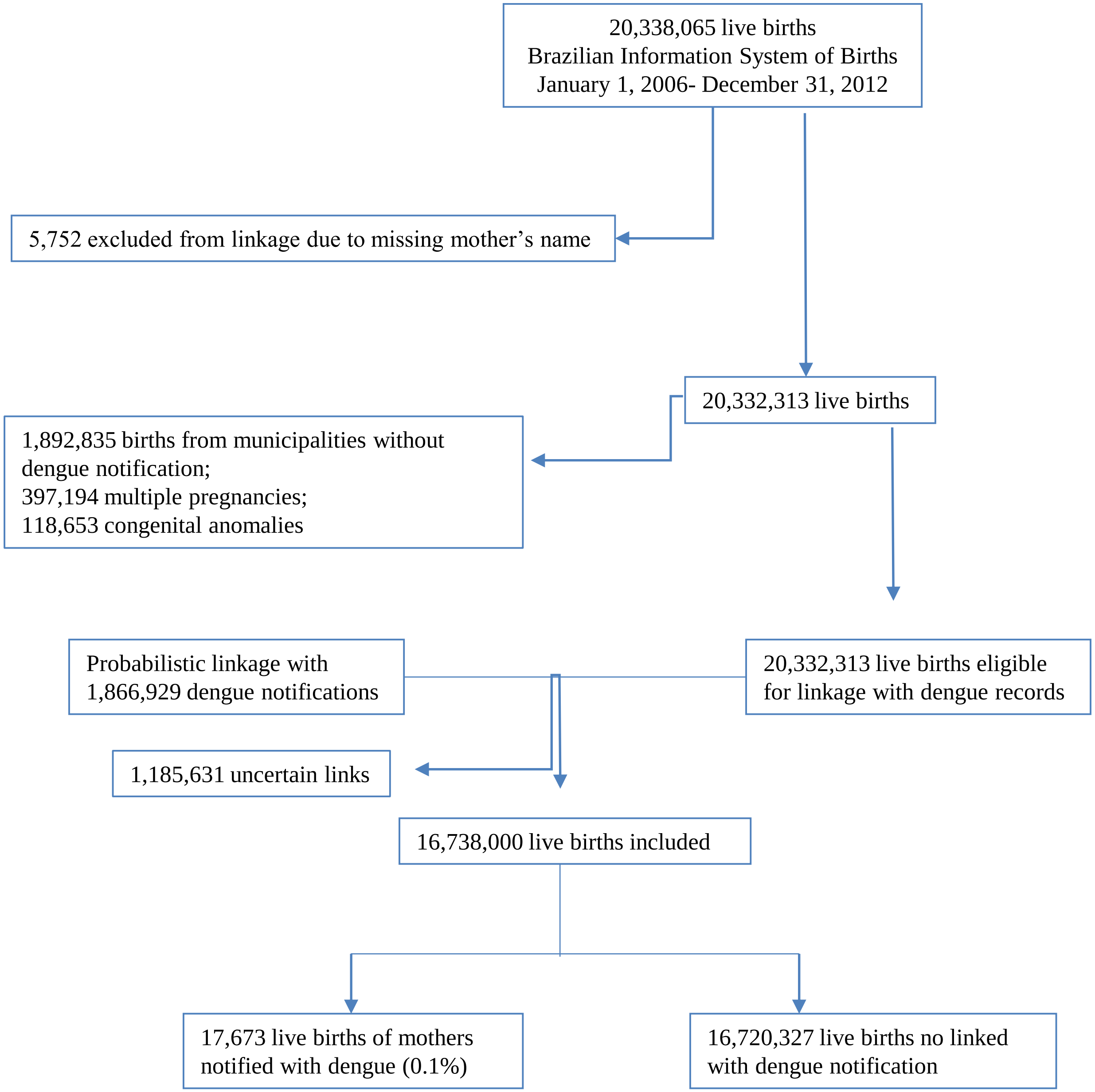

STROBE Statement—Checklist of items that should be included in reports of ***cohort studies***

	Item No	Recommendation
**Title and abstract**	1	(*a*) Indicate the study's design with a commonly used term in the title or the abstract-page 1
		(*b*) Provide in the abstract an informative and balanced summary of what was done and what was found- page 2
**Introduction**		
Background/rationale	2	Explain the scientific background and rationale for the investigation being reported-page 4
Objectives	3	State specific objectives, including any prespecified hypotheses- page 4
**Methods**		
Study design	4	Present key elements of study design early in the paper- page 5 paragraph 89-91
Setting	5	Describe the setting, locations, and relevant dates, including periods of recruitment, exposure, follow-up, and data collection- page 5 this is a linkage study of pregnant women, therefore there specific details described during the methods section.
Participants	6	(*a*) Give the eligibility criteria, and the sources and methods of selection of participants. Describe methods of follow-up-page 7 paragraph 152
		(*b*) For matched studies, give matching criteria and number of exposed and unexposed- N/A
Variables	7	Clearly define all outcomes, exposures, predictors, potential confounders, and effect modifiers. Give diagnostic criteria, if applicable- page6-8
Data sources/ measurement	8*	For each variable of interest, give sources of data and details of methods of assessment (measurement). Describe comparability of assessment methods if there is more than one group-page 6-8 and table 1
Bias	9	Describe any efforts to address potential sources of bias- page 8
Study size	10	Explain how the study size was arrived at- figure 1
Quantitative variables	11	Explain how quantitative variables were handled in the analyses. If applicable, describe which groupings were chosen and why- n/a
Statistical methods	12	(*a*) Describe all statistical methods, including those used to control for confounding-page 8
		(*b*) Describe any methods used to examine subgroups and interactions- page 8
		(*c*) Explain how missing data were addressed- page 8
		(*d*) If applicable, explain how loss to follow-up was addressed N/A
		(*e*) Describe any sensitivity analyses- page 8
**Results**		
Participants	13*	(a) Report numbers of individuals at each stage of study—eg numbers potentially eligible, examined for eligibility, confirmed eligible, included in the study, completing follow-up, and analysed- figure 1and page
		(b) Give reasons for non-participation at each stage-figure 1
		(c) Consider use of a flow diagram-figure 1 and page 9
Descriptive data	14*	(a) Give characteristics of study participants (eg demographic, clinical, social) and information on exposures and potential confounders-table 1page 9
		(b) Indicate number of participants with missing data for each variable of interest-table 1
		(c) Summarise follow-up time (eg, average and total amount)- n/a
Outcome data	15*	Report numbers of outcome events or summary measures over time- table 1 page 9-10

For peer review only

For peer review only - http://bmjopen.bmj.com/site/about/guidelines.xhtml

Main results	16	(*a*) Give unadjusted estimates and, if applicable, confounder-adjusted estimates and their precision (eg, 95% confidence interval). Make clear which confounders were adjusted for and why they were included- table 2-4
		(*b*) Report category boundaries when continuous variables were categorized-methods section page 8
		(*c*) If relevant, consider translating estimates of relative risk into absolute risk for a meaningful time period-n/a
Other analyses	17	Report other analyses done—eg analyses of subgroups and interactions, and sensitivity analyses- page 8
**Discussion**		
Key results	18	Summarise key results with reference to study objectives-page 13
Limitations	19	Discuss limitations of the study, taking into account sources of potential bias or imprecision. Discuss both direction and magnitude of any potential bias page 15
Interpretation	20	Give a cautious overall interpretation of results considering objectives, limitations, multiplicity of analyses, results from similar studies, and other relevant evidence-page 15
Generalisability	21	Discuss the generalisability (external validity) of the study results- page 15
**Other information**		
Funding	22	Give the source of funding and the role of the funders for the present study and, if applicable, for the original study on which the present article is based-page 19

*Give information separately for exposed and unexposed groups.

Note: An Explanation and Elaboration article discusses each checklist item and gives methodological background and published examples of transparent reporting. The STROBE checklist is best used in conjunction with this article (freely available on the Web sites of PLoS Medicine at http://www.plosmedicine.org/, Annals of Internal Medicine at http://www.annals.org/, and Epidemiology at http://www.epidem.com/). Information on the STROBE Initiative is available at http://www.strobe-statement.org.

For peer review only

For peer review only - http://bmjopen.bmj.com/site/about/guidelines.xhtml
